# Is Dental Work Expensive

**Published:** 1884-05

**Authors:** D. R. Stubblefield

**Affiliations:** Nashville, Tenn.


### IS DENTAL WORK EXPENSIVE.

BY D. R. STUBBLEFIELD, A. M., M. D., D. D. S., NASHVILLE, TENN.

Probably every practitioner has had this question directly or indirectly propounded to him. Indeed, in the mind of the average individual, it is a deep-seated conclusion that dental service, of all work, is the most dearly paid for.

It behooves us as men and members of an important branch of the science of human conservation, to set the facts in order, and no longer be misrepresented willfully or unintentionally, as the case may be. We have a right to demand a fair hearing, and we ought not to ask more than this.

The question may arise just here as to whether any man can impartially consider a case in which he is interested. Without delaying, I think there are some who can reach a state of almost perfect self-abnegation, and might be trusted. It is a question, though, which each may settle in his own mind, for we may not judge for others.

First, let us have a good understanding as to what we are considering, for argument can be conducted fairly only when the primary proposition is understood alike by both. If I discuss one thing and you another, we may have four sides complicating our discussion of a picture which should have but two sides. Good dental work is the only kind to be considered, because, as all will agree, imperfect—that is, bad dental work—is not worth any price. Everybody has a right to demand good dental work as a fair return for value received. But when good dental work has been given, is the price set upon it by a fair-minded dentist to be considered exorbitant? That is the question. Price is regulated, as economists say, by demand and supply. I have my case then, if I show that both, in this instance, conspire to justify even a high price for this kind of work.

The demand for anything depends upon the nearness which that thing approaches our necessities, or should intrinsically depend upon such a basis. Of course, this must be modified by the consideration of true and fictitious wants, and the degree with which a want is felt, for it may be imagined that a true want is not felt, and a fictitious want is very much felt. I have not time to elaborate this, but no one of fair mind will take this issue when I make no challenge, especially when I point it out myself. I merely wish to show that the want of dental work is a real one, whether felt or not, because of the importance of the teeth. On this I propose to base the argument for demand. Every doctor will tell you of the importance of thorough mastication, and any body knows just how important the teeth are for that purpose. You may cut up your food in small pieces, and you may even confine yourself to liquids, or semi-solids which may be easily swallowed, but you do not make any argument against the utility of the teeth and the importance of mastication. Along with mastication nature has designed that insalivation, or thorough admixture of food and saliva, shall take place, and this takes place most perfectly when mastication is most perfect. Diseases result, or when present are aggravated, by the presence of unmasticated food in the alimentary canal. To preserve these organs then, or to repair them when invaded by hurtful agencies, is an important work.

Here is another and less positive way to prove the importance of the teeth, or, rather, their value: Ask the first man you meet who has good teeth, what he would take for the four front ones, and what do you suppose he would say? Depending upon the condition of his stomach and pocket-book, he would say from five dollars to fifty thousand dollars. But how much would you, Mr. I. P., say yours were worth each? Fifty dollars? that's fourteen hundred dollars for twenty eight; five hundred dollars? that's fourteen thousand dollars for that number. As you are able to live, money could not buy them, I am sure.

Good teeth are blessings which cannot be too highly estimated for health, or for beauty's sake. And remember they, like other blessings, brighten as they take their flight. Artificial teeth! Their very existence proves my proposition; and they, at their best, are but judicious imitations, which rarely, if ever, equal their natural predecessors. In fine, the demand is incontestible.

Now for the supply to meet this demand. Good dental work cannot be done repeatedly by a bad dentist. I make no provision for the occasional or accidental work—bad by a good dentist, and good by a bad dentist—for in the one case you can get redress by the showing, and in the other no positive skill is displayed. I say good dental work can be done repeatedly only by a good dentist, because it requires skill and attainments which only such have achieved. Every workman in any calling is not as skillful as the best in that calling. In fact, a number one man in any is the exception and not the rule. Apply this to dentistry. Every man who has the name of dentist cannot do the highest type of dental work—that type which you have a right to demand—just as every man who can drive a nail is not a first-class carpenter. There are grades among dentists, just as there are in any calling in life; and the tree that brings forth good fruit continually is the good tree, and *vice-versa.*

As, then, good dentists are the exception and not the rule, if there is any demand (and we have shown there is) for good dental work, the supply cannot be so plentiful as to glut the market.

In this connection I may say the prices for the best work in various pursuits differ as these pursuits differ. For instance, a woodcut artist is not paid so well as an engraver of bank notes, and you would not think of paying as much for treating your cart-horse as for treating your thorough-bred colt.

Now, mind you, we suppose in every case that the service is satisfactory, and as such is entitled to the best price for such work.

Good dental work, which you have a right to demand, stops the destruction of the teeth, as a rule, and preserves these important organs for long years, if not a lifetime of usefulness. As such it ought to be paid for equivalently and equitably, even if it does seem at the time to take a good many dollars. Say to your patients, get none but the best dentist and demand his best skill, and you practice economy. Get one on whom you can rely, take his time and skill (that, if anything, he should be paid for), and then do not question his bill and wound his feelings, for you only make yourself small by so doing. And when at the meridian of life you still have the ability to save your stomach much hard work by proper chewing, and your health many a midnight inroad of acute indigestion, give your dentist some credit when you applaud your own good judgment for your early wisdom.

